# Poly(2-hydroxyethyl methacrylate)-quercetin Conjugate as Biomaterial in Ophthalmology: An “*ab initio*” Study

**DOI:** 10.3390/jfb2010001

**Published:** 2011-01-27

**Authors:** Manuela Curcio, Giuseppe Cirillo, Ortensia Ilaria Parisi, Francesca Iemma, Umile Gianfranco Spizzirri, Ilaria Altimari, Nevio Picci, Francesco Puoci

**Affiliations:** 1 Dipartimento di Scienze Farmaceutiche, Università della Calabria, Edificio Polifunzionale, 87036 Arcavacata di Rende (CS), Italy; E-Mails: manuela.curcio@unical.it (M.C.); ortensiailaria.parisi@unical.it (O.I.P.); francesca.iemma@unical.it (F.I.); g.spizzirri@unical.it (U.G.S.); ilaria.altimari@unical.it (I.A.); nevio.picci@unical.it (N.P.); francesco.puoci@unical.it (F.P.); 2 Macrofarm Srl, Università della Calabria, Edificio Polifunzionale, 87036 Arcavacata di Rende (CS), Italy

**Keywords:** poly(2-hydroxyethyl methacrylate, quercetin, ocular tolerance, polymeric antioxidant

## Abstract

A novel polymeric material with antioxidant properties and suitable for ophthalmic application was synthesized by free radical grafting reaction between 2-hydroxyethyl methacrylate and quercetin. The presence of quercetin in the polymeric chain was confirmed by FT-IR and UV-Vis analyses, while an estimation of the amount of quercetin bound per gram of polymer was obtained by the Folin-Ciocalteu assay. The conjugate shows high biocompatibility (HET-CAM assay) and antioxidant and anti-inflammatory properties which were extensively investigated by specific *in vitro* tests.

## 1. Introduction

The synthesis of biocompatible, comfortable and durable materials to be used in ophthalmology is still a challenge. Synthetic hydrogels occupy an important position among biomaterials due to their hydrophilicity and ability to absorb water without losing their viscoelastic behavior [1]. Poly(2-hydroxyethyl methacrylate) (pHEMA) was one of the first components of hydrogels and is still widely used due to its biomimetic properties. Furthermore, the mechanical features of pHEMA hydrogels can be easily modulated through co-polymerization with co-monomers and cross-linking agents of different natures. Thus, pHEMA is still a strong candidate for the development of flexible hydrogel to be used in ophthalmology and for new medicated soft contact lenses in particular [2,3].

Contact lenses (CL) are increasing in popularity as new materials and efforts are made to render them easier to fit and more comfortable to wear [4]. Medicated contact lenses (CL) are attracting escalating interest because of their ability to prolong the residence time of drugs on the precorneal area, increasing the ocular bioavailability and diminishing loss by blinking and unproductive systemic absorption [5].

CL have a wide variety of effects on the cornea attributable to their mechanical effect and to their tendency to impair oxygen delivery to the cornea [6]. Since the epithelium derives virtually all of its oxygen from the atmosphere, CL have the capability of interrupting that supply [7]. The cornea is particularly at risk from oxidative damage, owing to direct light exposure and environmental insults such as air pollutants and ultraviolet (UV) radiation [8]. The free radical and reactive oxygen species (ROS) generation can trigger corneal damage owing to potential modifications of proteins, lipids, and DNA [9]. Oxidative stress is involved in many ocular diseases such as age-related macular degeneration, retinopathy of prematurity, retinal light damage, and cataract [10,11].

The definition of oxidative stress implies increased oxidant production and/or a decreased antioxidant capacity in animal cells characterized by the release of free radicals, resulting in cellular degeneration [12]. This has been supported by the protective effect of physiologic antioxidants, like pyruvate, and nutritional antioxidants, such as ascorbate, vitamin E and carotenoids [13]. During the past two decades, much attention has been paid to the possible roles of physiologic and nutritional antioxidants in ocular diseases [14]. Among these, special attention has been paid to plant polyphenols. As efficient free radical scavengers, they can potentially interact with biological systems and play a role in preventing human neurodegenerative diseases, cardiovascular disorders and cancer [15,16]. Phenolics display a wide variety of structures, ranging from simple moieties containing a single hydroxylated aromatic ring to highly complex polymeric substances. Based on their carbon skeleton, polyphenols can be classified into non-flavonoid compounds and flavonoid compounds [17]. Many studies have been carried out with quercetin and (epi) catechin, as they are major flavonoids in the human diet that are easily available as commercial standards. These compounds behave as powerful antioxidants and free radical scavengers [18] and are able to interact with several key enzymes [19].

In recent years, an emerging research field concerns the covalent insertion of antioxidants in polymeric chains [20]. They could be applied in those fields in which the employment of a single molecule with antioxidant activity is prohibitive [21]. Functionalization with antioxidant molecules is a promising approach to improve the performance of natural polymers, opening new applications in the biomedical and pharmaceutical fields. The resulting antioxidant-polymer conjugates, combining the advantages of both the components, shows a higher stability and slower degradation rate than molecules with a low molecular weight, but preserves the unique properties of antioxidant molecules [22]. In literature, several approaches were explored for the preparation of biopolymers coupled with antioxidant compounds; in this work, the adopted synthetic strategy is the grafting reaction between the antioxidant molecule and the growing polymeric chain in a single-step reaction. As reported in recent papers, this approach is very useful to synthesize polymer-antioxidant conjugates without the generation of toxic reaction by-products and, at room temperatures, preserving the antioxidant by degradation processes [23]. In this works, the synthetic approach was adopted to prepare a new pHEMA-antioxidant conjugate which was extensively investigated in terms of antioxidant, anti-inflammatory and biocompatibility properties.

For the synthesis of this conjugate, Quercetin (Q) was selected as antioxidant because of its high antioxidant and anti-inflammatory properties which make this flavonoid of great interest for biomedical applications [24,25]. Because of its properties, Q was also used in ophthalmology to prevent the cataract formation in *in vitro* models [26]. Based on these considerations, Q represents an ideal candidate for the synthesis of a new generation of HEMA conjugates.

## 2. Experimental Section

### 2.1. Materials

2-hydroxyethylmetacrylate (HEMA), 2,2′-azoisobutyronitrile (AIBN), quercetin (Q), hydrogen peroxide (H_2_O_2_), ascorbic acid (AA), 2,2′-diphenyl-1-picrylhydrazyl radical (DPPH), Folin-Ciocalteu Reagent (FCR), β-carotene, linoleic acid, fluorescein, ammonium molybdate, deoxyribose, Tween 20, sodium nitrite, sodium carbonate, disodium hydrogen phosphate, sodium dihydrogen phosphate, trisodium phosphate, manganese oxide (MnO_2_), FeCl_3_, ethylenediaminetetraacetic acid disodium salt (EDTA), dipotassium hydrogen phosphate, potassium dihydrogen phosphate, thiobarbituric acid (TBA), trichloroacetic acid (TCA), sulphuric acid (96% w/w), hydrochloridc acid (37% w/w), sodium potassium tartrate, sodium hydroxide, lithium bromide and polymethylmethacrylate (pMMA) standard samples for size-exclusion chromatography (M_n_ = 520–811,000; M_w_/M_n_ = 1.05–1.28) were obtained from Sigma-Aldrich (Sigma Chemical Co., St Louis, MO, U.S.). N,N-dimethylformamide (DMF), water and ethanol were HPLC-grade and provided by Carlo Erba reagents (Milan, Italy).

### 2.2. Instruments

The liquid chromatography consisted of a Jasco PU-2089 Plus liquid chromatography apparatus equipped with a Rheodyne 7725i injector (fitted with a 20 μL loop), a Jasco UV-2075 HPLC detector and Jasco-Borwin integrator. A reverse-phase C18 Hibar column, 250 mm × 4 μm, particle size = 5 μm, pore size = 120Å (Merck, Darmstadt, Germany), was employed. According to literature data, the mobile phase adopted for the detection of quercetin [27] was a 1% (v/v) formic acid aqueous solution-acetonitrile-2-propanol (70:22:8) at a flow rate of 0.2 mL min^−1^. Chromatograms were recorded at 370 nm.

For pHEMA and pHEMA-Q, Mn and Mw/Mn were measured by size-exclusion chromatography (SEC) using DMF containing 10 mM LiBr as eluent at 40 °C on three polystyrene-gel columns (Shodex K-805 L, Shodex, Tokyo, Japan (pore size: 20–1000 A; 8.0 mm i.d. × 30 cm); flow rate, 1.0 mL min^−1^) connected to a Jasco Jasco PU-2089 pump and a Jasco 930-RI refractive-index detector. The columns were calibrated standard pMMA samples.

IR spectra were recorded as KBr pellets on a Jasco FT-IR 4200. The dialysis membranes of 6-27/32″ Medicell International Ltd. (MWCO: 12-14000 Da) were employed. Freeze drier Micro Modulyo, Edwards was employed.

UV-Vis absorption spectra were obtained with a Jasco V-530 UV/Vis spectrometer.

### 2.3. Synthesis of pHEMA-Q Conjugate

The polymerization of HEMA with Quercetin (Q) was carried out as follows: in a 10 mL glass tube, 0.1; 0.25; 0.5 g of Q were dissolved in 8.0 mL of DMF and then HEMA (5.0 g) and 100 mg of AIBN were added and the solution was magnetically stirred at 60 °C. After 24 h, the mixture was poured into a non-solvent (acetone), and then the precipitated polymer was filtered by sintered glass filter funnel (Pyrex, Ø30 mm; porosity 3), submitted to three dissolution/precipitation cycles (methanol/acetone), and vacuum-dried at room temperature.

The polymer was checked to be free of unreacted Q and any other compounds by HPLC analysis after each purification step.

Blank polymer (pHEMA) was prepared under the same conditions without using Q.

### 2.4. Ocular Tolerance Test (HET-CAM Test)

HET-CAM was performed as an alternative *in vitro* eye irritation method to the one described in literature [28]. This test is ideal to evaluate the potential irritation (PI) effects in the eye (hemorrhage, vasoconstriction, and coagulation) caused after 5 min application of the product over the chorioallantoic membrane (CAM) of chicken egg. This membrane is well irrigated and has a similar structure to the irrigated eye tissues, like the conjunctive. Fertilized eggs were obtained from a poultry farm. Three eggs for each formulation weighing between 50 and 60 g were selected and candled to discard the defective ones. Prior to the experiment, the fertilized chicken eggs were incubated for 10 days in controlled conditions (37.8 °C and 50–60% RH). The trays containing eggs were rotated manually in a gentle manner every 12 hours. The shell and the first whiteness membrane were removed after wetting with an isotonic NaCl solution at 37 °C. Then 0.5 mL of pHEMA-Q suspensions (concentrations ranging from 1.0 mg mL^−1^ to 50.0 mg mL^−1^) in water was applied over the CAM and eventually eye irritation effects were observed. To afford a semi-quantitative evaluation of the reactions, preliminary tests with reference substances were performed applying isotonic NaCl solution, Texapon®, sodium sodium dodecyl sulfate (SDS, 1%) and sodium hydroxide (0.1 N) onto CAMs, in order to distinguish between none, weak, moderate and severe effects [29]. The scores were recorded according to the scoring schemes as shown in Table 1.

**Table 1:** Scoring chart for HET-CAM test.

**Effect**	**Score**	**Inference**
No visible hemorrhage	0	Non irritant
Just visible membrane discoloration	1	Mild irritant
Structures are covered partially due to membrane discoloration or hemorrhage	2	Moderately irritant
Structures are covered totally due to membrane discoloration or hemorrhage	3	Severe irritant

### 2.5. Evaluation of Disposable Phenolic Groups by Folin-Ciocalteu Procedure

Amount of total phenolic equivalents was determined using the Folin-Ciocalteu reagent procedure, according to the literature with some modifications [30]. Briefly, 20 mg of pHEMA-Q conjugate was dispersed in distilled water (6 mL) in a volumetric flask. The Folin-Ciocalteu reagent (1 mL) was added and the contents of the flask were mixed thoroughly. After 3 min, 3 mL of Na_2_CO_3_ (2%) was added, and then the mixture was allowed to stand for 2 h with intermittent shaking. The absorbance was measured at 760 nm against a control prepared using the blank polymer under the same reaction conditions. The amount of total phenolic groups in the polymeric material was expressed as quercetin equivalent (mg) by using the equation obtained from the calibration curve of the free antioxidant, recorded by employing five different Q standard solutions. 0.5 mL of each solution were added to the Folin-Ciocalteu system to raise the final concentration of 8.0, 16.0, 24.0, 32.0, and 40.0 × 10^−6^ mol L^−1^, respectively. After 2 h, the absorbance of the solutions was measured to record the calibration curve and the correlation coefficient (*R*^2^), slope and intercept of the regression equation obtained were calculated by the method of least squares.

### 2.6. Hydrophilic Properties of Polymers

The hydrophilic properties of pHEMA and pHEMA-Q were determined as follows. Briefly, aliquots (40–50 mg) of the polymers dried to constant weight, were placed in a 5-mL sintered glass filter (Ø10 mm; porosity, G3), weighted and left to swell by immersing the filter plus support in a beaker containing the swelling media (PBS solution, pH = 7.0, at 37 °C). After 24 h, the excess of water was removed by percolation at atmospheric pressure. Then, the filter was placed in a properly sized centrifuge test tube by fixing it with the help of a bored silicone stopper, then centrifuged at 3500 rpm for 15 min and weighted. The filter tare was determined after centrifugation with only water. The weights recorded at the different times were averaged and used to give the water content percent (WR%) by the following Equation (1):

(1) $$WR\%=\frac{W_{s}-W_{d}}{W_{d}}\times 100$$

### 2.7. Determination of Scavenging Effect on the DPPH Radical

In order to evaluate the free radical scavenging properties of pHEMA-Q conjugate, its reactivity towards a stable free radical, 2,2′-diphenyl-1- picrylhydrazyl radical (DPPH), was evaluated [31]. For this purpose, in each of seven test tubes, 5.0, 10.0, 15.0, 20.0 mg amounts of pHEMA-Q conjugate were dissolved in 6 mL of ethanol and then 4 mL of ethanol solution of the DPPH (250 × 10^−6^ mol L^−1^) were added, obtaining a solution of the DPPH with a final concentration of 100 × 10^−6^ mol L^−1^. The sample was incubated in a water bath at 25 °C and, after 30 min, the absorbance of the remaining DPPH was determined colorimetrically at 517 nm. The same reaction conditions were applied on the blank pHEMA and quercetin standard solutions (positive control). The scavenging activity of the tested polymeric materials was measured as the decrease in the absorbance of the DPPH and it was expressed as percent inhibition of the DPPH radical calculated according to the following Equation 2:

(2) $$\text{inhibition}\left(\%\right)=\frac{A_{0}-A_{1}}{A_{0}}\times 100$$

where A_0_ is the absorbance of a standard prepared in the same conditions, but without any polymers, and A_1_ is the absorbance of polymeric samples.

### 2.8. Determination of Total Antioxidant Activity

The total antioxidant activity of polymeric materials was evaluated according to the method reported in literature [32]. Briefly, 20 mg of pHEMA-Q conjugate were mixed with 2.4 mL of reagent solution (0.6 mol L^−1^ sulphuric acid, 28 mol L^−1^ trisodium phosphate and 4 mol L^−1^ ammonium molybdate) and 0.6 mL of methanol, then the reaction mixture was incubated at 95 °C for 150 min. After cooling to room temperature, the absorbance of the mixture was measured at 695 nm against a control prepared using blank polymer in the same reaction condition. The total antioxidant activity was expressed as Q equivalent (mg). By using five different Q standard solutions, a calibration curve was recorded. 0.3 mL of each solution were mixed with 1.2 mL of reagent solution to obtain the final concentration of 8.0, 16.0, 24.0, 32.0, and 40.0 × 10^−6^ mol L ^−1^, respectively. After 150 min incubation, the solutions were analyzed by UV-Vis spectrophotometer and the correlation coefficient (*R*^2^), slope and intercept of the regression equation obtained by the method of least squares were calculated.

### 2.9. β-Carotene-Linoleic Acid Assay

The antioxidant properties of synthesized functional polymer were evaluated through measurement of percent inhibition of peroxidation in linoleic acid system by using the β-carotene bleaching test [33]. Briefly, 1 mL of the β-carotene solution (0.2 mg mL^−1^ in chloroform) was added to 0.02 mL of linoleic acid and 0.2 mL of Tween 20. The mixture was then evaporated at 40 °C for 10 min in a rotary evaporator to remove chloroform. After evaporation, the mixture was immediately diluted with 100 mL of distilled water. The water was added slowly to the mixture and agitated vigorously to form an emulsion. The emulsion (5 mL) was transferred to different test tubes containing 2.0; 3.0; 4.0; 5.0 mg L^−1^ concentration of pHEMA-Q conjugate. The tubes were then gently shaken and placed in a water bath at 45 °C for 60 min. The absorbance of the filtered samples and control was measured at 470 nm against a blank, consisting of an emulsion without the β-carotene. The measurement was carried out at the initial time (*t* = 0) and successively at 60 min intervals. The same reaction conditions were applied by employing pHEMA in order to evaluate the interference of polymeric material on the bleaching assay and quercetin standard solutions (acting as a positive control). The antioxidant activity (A*_ox_*A) was measured in terms of successful bleaching of the β-carotene using the following Equation (3):

(3) $$A_{ox}A=\left(1-\frac{A_{0}-A_{60}}{A_{0}^{0}-A_{60}^{0}}\right)$$

where A_0_ and A_0_° are the absorbance values measured at the initial incubation time for samples and control, respectively, and A*_60_* and A*_60_*° are the absorbance values measured in the samples and in control, respectively, at *t* = 60 min.

### 2.10. Determination of Scavenging Properties on Peroxynitrite Anion

Peroxynitrite was synthesized from sodium nitrite/H_2_O_2_, acidified with HCl and the residual H_2_O_2_ was removed by passing the solution through granular MnO_2_. The yellowish stock solution was stored at −80 °C and its concentration was evaluated immediately before its use by measuring the absorbance at 302 nm. The measurements of relative antioxidant capacity were determined by using fluorescein as detecting molecule. Briefly, Fluorescein (to obtain 2 × 10^−6^ mol L^−1^ final) in 100mM phosphate buffer, pH 7.4, was mixed in the presence or absence of antioxidant with 100 × 10^−6^ mol L^−1^ ONOO^−^ to the final volume of 2 mL. The tested concentrations of pHEMA-Q conjugate were 1.25; 2.50; 3.75 mg mL^−1^. As reported in literature, the immediate mixing of the sample with the oxidant added is critical for the reproducibility of the assay [34]. After incubation at room temperature for 15 min, the fluorescence of the samples was measured (excitation: 485 nm, emission: 538 nm).

The same reaction conditions were applied on the pHEMA in order to evaluate the activity of polymeric material and on quercetin standard solutions (positive control).

### 2.11. Scavenging Activity on Hydroxyl Radical

The scavenging activity on hydroxyl radical was evaluated according to the literature [35]. Briefly, in four different test tubes, 5.0; 10.0; 15.0; 20.0 mg of pHEMA-Q were dispersed in 0.5 mL of 95% ethanol and incubated with 0.5 mL deoxyribose (3.75 mM), 0.5 mL H_2_O_2_ (1 mM), 0.5 mL FeCl_3_ (100 mM), 0.5 mL EDTA (100 mM) and 0.5 mL ascorbic acid (100 mM) in 2.0 mL potassium phosphate buffer (20 mM, pH 7.4) for 60 min at 37 °C (Pan *et al.* 2007). Then samples were filtered and to 1 mL amount of filtrate, 1 mL of TBA (1% w/v) and 1 mL of TCA (2% w/v) were added. and the tubes were heated in a boiling water bath for 15 min. The content was cooled and the absorbance of the mixture was read at 535 nm against reagent blank without Q. The same reaction conditions were applied on the pHEMA in order to evaluate the interference of polymeric material and on free quercetin acting as positive control.

### 2.12. Anti-Inflammatory Effect

NO generated from sodium nitroprusside (SNP) was measured according to the method reported in literature [36]. Briefly, the reaction mixture (5.0 mL) containing SNP (5 mM) in phosphate-buffered saline (pH 7.3), with or without the polymers at concentrations of 1.5; 3.0; 4.5; 6.0 mg mL^−1^, was incubated at 25 °C for 180 min in front of a visible polychromatic light source (25 W tungsten lamp). The NO^•^ radical thus generated, interacted with oxygen to produce the nitrite ion (NO_2_^−^) which was assayed at 30 min intervals by mixing 1.0 mL of incubation mixture with acid and an equal amount of Griess reagent (1% sulfanilamide in 5% phosphoric 0.1% naphthylethylenediamine dihydrochloride). The absorbance of the chromophore (purple azo dye) formed during the diazotisation of nitrite ions with sulphanilamide and subsequent coupling with naphthylethylenediamine dihydrochloride was measured at 546 nm. The antioxidant activity was expressed as a percentage of scavenging activity according to Equation (2).

The same reaction conditions were applied on free quercetin acting as a positive control.

### 2.13. Statistical Analyses

Each measurement was carried out in five independent experiments; data were expressed as means (± SD), and analyzed using one-way analysis of variance (ANOVA). For the inhibitory experiment, the IC50 value was determined as the concentration of an antioxidant that provides 50% inhibition.

## 3. Results and Discussion

### 3.1. Synthesis and Characterization of the pHEMA-Q Conjugate

Among the several synthetic strategies which are proposed in the literature for the synthesis of polymer-antioxidant conjugates [37], the free radical grafting procedure was recently explored as a new useful methodology for the synthesis of polymeric antioxidants, both synthetic and natural [38]. In this work, this technique was applied in the synthesis of the pHEMA-Q derivative with antioxidant and anti-inflammatory activities.

The synthesis of the conjugate was carried out according to literature data [38]. 0.5 mg of quercetin per 5.0 mg of HEMA corresponds to the highest amount of quercetin which can be dissolved in the reaction medium. A lower amount of Q in the reaction mixture led to less effective conjugates as recorded by the Folin-Ciocalteu assay. Literature data suggest that the insertion of aromatic antioxidants on the polymeric backbone occurs in the ortho- and para-positions relative to the hydroxyl group [39]. To remove un-reacted antioxidant physically incorporated in the polymeric structure, the conjugates underwent precipitation-dissolution cycles and the non-solvents were analyzed by HPLC.

The molecular weight distributions of synthesized polymers were analyzed by a SEC, and the results show an average molecular weight of 92,000 Da and 81,000 Da for pHEMA and pHEMA-Q, respectively with M_w_/M_n_ < 1.5.

The covalent incorporation of quercetin in the final polymer was confirmed by performing FT-IR and UV-Vis analyses, while a crude quantitative determination of the amount of quercetin bound per g of polymeric material was obtained by the Folin-Ciocalteu assay.

**Figure 1:**
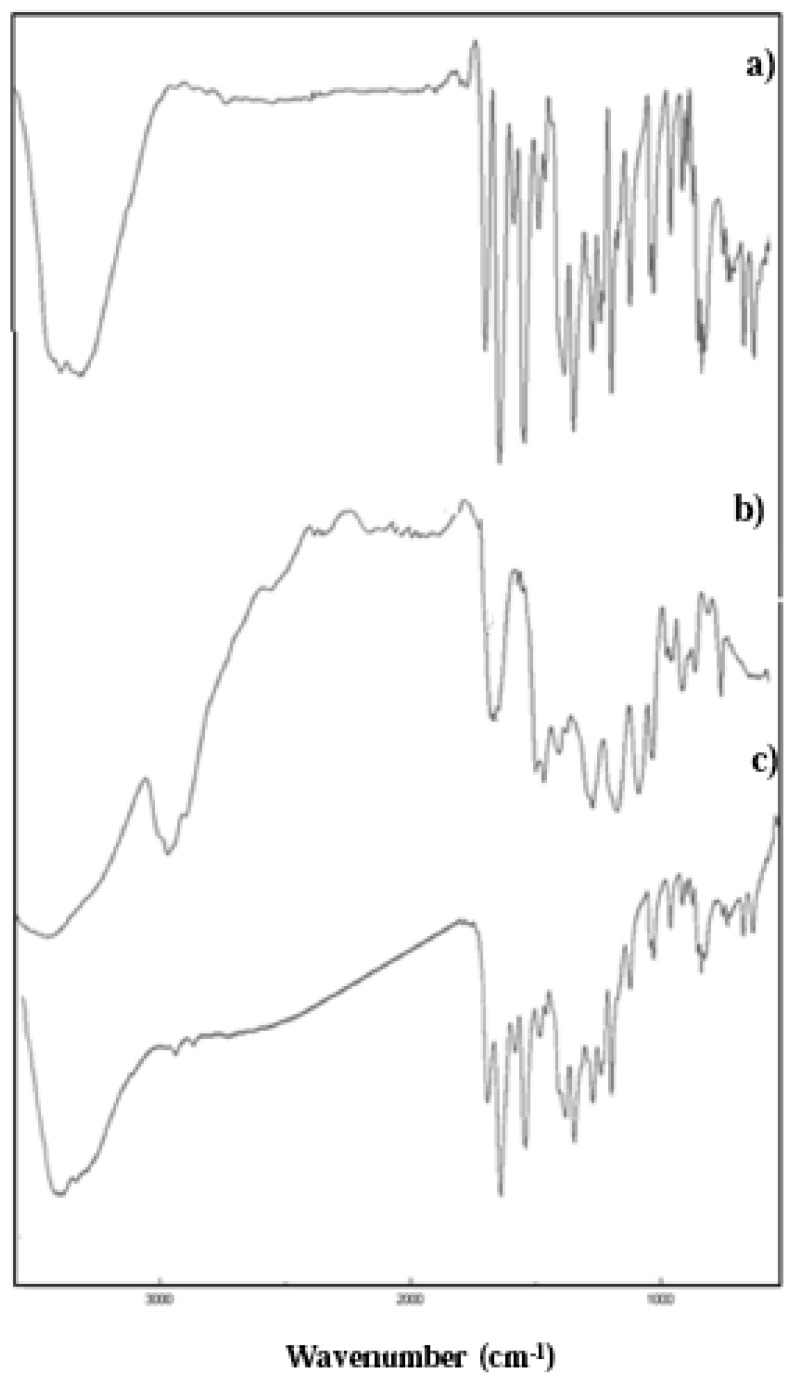
FT-IR spectra of **(a)** Q; **(b)** pHEMA and **(c)** pHEMA.

FT-IR spectra of pHEMA-Q conjugate show the appearance of the peaks attributable to the presence of quercetin: in particular the peaks at 1515 cm^−1^ ascribable to carbon-to-carbon stretching within the aromatic ring (Figure 1).

**Figure 2:**
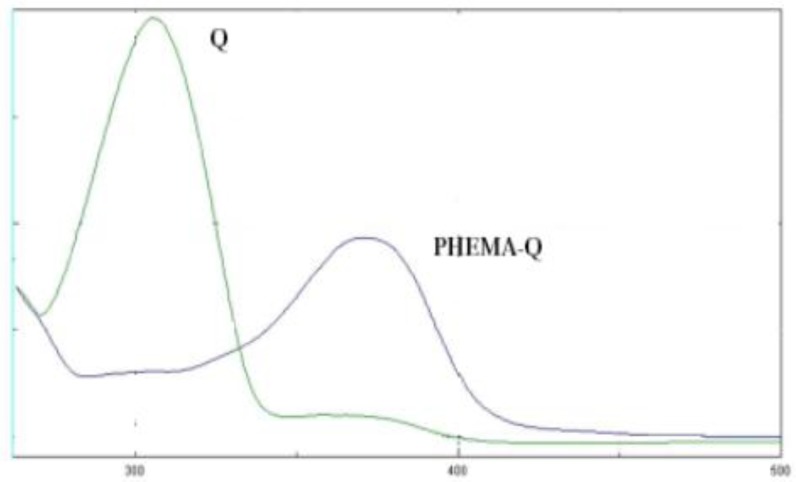
UV-Vis spectra of Q and pHEMA-Q.

A further confirmation of antioxidant insertion in the polymer was obtained by comparing the UV absorption spectra of free and bound antioxidant (Figure 2). A bathochromic shift of the absorption peak of Q was observed from 310 nm in the free form to 370 nm in the pHEMA-Q, and this red shift was used as a confirmation of the covalent linkage between the antioxidant and the polymeric chain of pHEMA-Q because no absorption peak was detected in the same wavelength range for blank pHEMA.

The hydrophilic properties of the materials, a parameter of great importance for ophthalmic applications, were examined and expressed as water content (WR%) after 24 h incubation in PBS solution (pH 7.0). The obtained data were 51 ± 1.2% for pHEMA and 59 ± 1.6 % for pHEMA-Q confirmed the good water affinity of the materials.

Another key point in the characterization of the obtained macromolecular system is the determination of its biocompatibility. As reported in literature, the main goal of toxicological scientific endeavors is to safeguard human beings against the possible adverse effects of diverse types of chemicals, including pharmaceuticals, cosmetics, household products, industrial chemicals, and agrochemicals [40]. No other field in *in vitro* toxicology testing has driven academic, industrial, and government resources to develop cell modeling systems as much as the need for alternatives to local toxicity testing [41,42]. Among the different *in vitro* tests, HET-CAM was emerged as an alternative *in vivo* approach for the testing of biomaterials, especially those to be applied in ophthalmology [43]. In this method, the evaluation of the biomaterial is performed by using the chorioallantoic membrane (CAM) of a developing chicken embryo that allows continuous visualization of the implant site while providing a rapid, simple, and low-cost screening of tissue reactions to biomaterials. The HET-CAM, thus, is a suitable alternative to animal testing, and it is based on the direct application onto the choriollantoic membrane of the sample and reactions, such as hemorrhage, intravasal coagulation or lysis of blood vessels are assessed on a time-course basis. These irritancy effects may occur within 5 min following mucosal administration of sample. The CAM is a non-innervated complete tissue containing arteries, veins and capillaries, and it is technically easy to study. It responds to injury with an inflammatory process similar to what one would observe in the conjunctival tissue of a rabbit's eye. Its well-developed vascularization provides an ideal model for ocular irritation studies [44].

**Figure 3:**
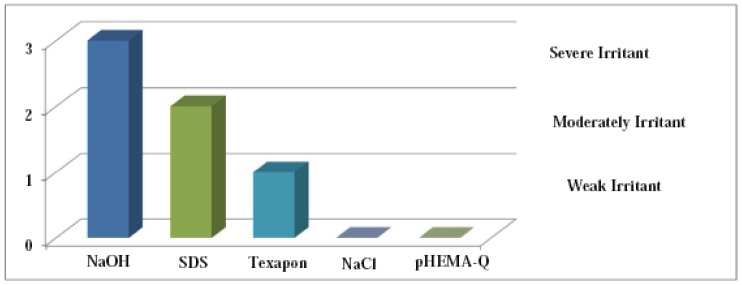
*In vitro* mean potential irritation results and classification of comparators and positive controls.

In a model protocol, to objectify response evaluation, Texapone ASV, sodium hydrochloride and acetic acid were tested in parallel for reference which enabled differentiation between none, weak, moderate and severe reactions of the possible endpoints hemorrhage, coagulation and vessel lysis (Figure 3).

**Figure 4:**
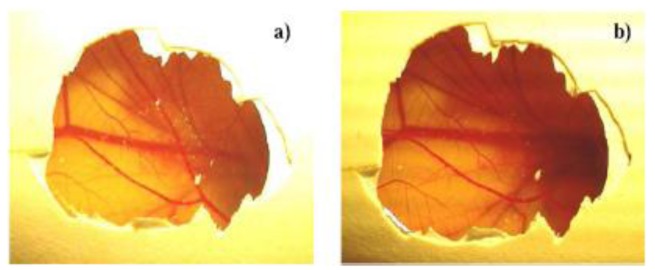
Photo documentation of the pHEMA-Q biocompatibility by HET-CAM test. **(a)** Untreated CAM. **(b)** CAM after the application of pHEMA-Q suspension in water (1.0 mg mL^−1^) for 5 min.

The CAM-morphology is showed in Figure 4a, while Figure 4b presents the CAM-morphology after 5 min treatment with pHEMA-Q. No signs of ocular irritancy have been detected by employing pHEMA-Q suspension at concentrations between 1.0 and 50.0 mg mL^−1^, as a consequence, an irritancy index of zero was assessed to the synthesized polymeric antioxidant, confirming that the proposed macromolecular system is non-irritant and well tolerated. The test was repeated in triplicate and no differences in the scoring chart were detected.

The whole of the antioxidant properties of the conjugate is ascribable to the presence of Q in the polymeric chain, and thus a useful characterization is the determination of the amount (mg) of quercetin bound per unit of mass (g) of conjugate. This value was calculated by the determination of the disposable phenolic content by the Folin-Ciocalteu assay. Folin-Ciocalteu reagent (FCR) contains a molybdenum complex which is reduced via electron-transfer reaction by the polyphenol with the subsequent formation of Mo(V) blue species (PMoW_11_O_40_)^4−^ spectrophotometrically detectable [30]. This assay was used as a crude determination of the amount of quercetin in the polymer by comparison with a quercetin calibration curve. The functionalization degree was found to be 1.46 ± 0.1 mg of quercetin per g of polymer.

By considering conjugates characterized by a lower amount of Q in the reaction mixture, this value significantly decreases, clearly proving that the ratio of 0.5 mg of Q per 5.0 g of HEMA represents the optimized condition to obtain the most effective antioxidant conjugate.

### 3.2. Evaluation of the Antioxidant and Anti Inflammatory Properties

The antioxidant properties of the synthesized pHEMA-Q were evaluated by specific antioxidant tests. First of all, the total antioxidant activity was evaluated by the molybdate assay [32]. The assay is based on the reduction of Mo(VI) to Mo(V) by the flavonoid in the polymer with the subsequent formation of a green phosphate/Mo(V) complex at acid pH. The total antioxidant activity was measured by comparison with the free antioxidant and the control pHEMA, which contained no antioxidant component. The high absorbance values indicated that the sample possessed significant antioxidant activity, which was expressed as mg equivalent of quercetin per g of dry polymer, and this value was found to be 1.02 ± 0.1 mg.

Ocular inflammation is the key target of the synthesized polymeric antioxidant. At the site of inflammation in the airway, mitogen-activated cells, such as macrophages, neutrophils, eosinophils, and epithelial cells, synthesize and release various pro-inflammatory mediators. Prostaglandin E2 (PGE2) and nitric oxide (NO•) are two pivotal pro-inflammatory mediators [45]. Despite the possible beneficial effects of NO•, its contribution to oxidative damage is increasingly becoming evident. This is due to the fact that NO• can react with superoxide to form the peroxynitrite anion, which is a potential strong oxidant that can decompose to produce •OH and NO_2_ [36]. SNP was used as an NO radical donor to evaluate the NO• scavenging activity of the pHEMA, and the IC_50_ value was found to be 3.94 ± 0.4 mg mL^−1^ (pHEMA was found to be ineffective), confirming the high potential applicability of the materials. This value, considering the results of the Folin-Ciocalteu assay, corresponds to a quercetin concentration of 4.87 μg mL^−1^. This value is quite similar to the IC_50_ of free quercetin which was found to be 4.19–4.87 μg mL^−1^, confirming that the conjugation process did not negatively interfere with the anti-inflammatory properties of the flavonoid.

After this characterization, the scavenging properties of the conjugate were tested. In particular, the scavenging activities against the lipophilic radical DPPH, hydroxyl radical (•OH), peroxynitrite anion (ONOO^−^) were determined and the ability of the conjugate to inhibit the lipid peroxidation evaluated.

**Figure 5:**
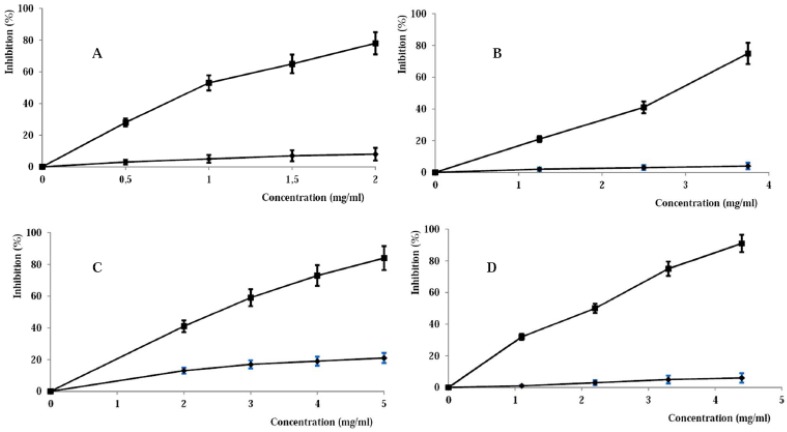
Inhibition profile on DPPH radical (**A**), peroxynitrite anion (**B**), peroxyl radical (**C**) and hydroxyl radical by pHEMA-Q (- -■- -) and pHEMA (—♦—). Data are expressed as means (± SD) of five independent experiments.

DPPH• is one of the few stable and commercially available organic nitrogen radicals with an absorption maximum band of around 515–528 nm. The DPPH assay has become quite popular in antioxidant studies because this method is simple and highly sensitive. DPPH• accepts an electron or hydrogen radical to become a stable molecule, and thus the antioxidant effect is proportional to the disappearance of DPPH• in test samples [31]. From the DPPH• inhibitory profile (Figure 5A) of pHEMA and pHEMA-Q, the high antioxidant power of the conjugate is clear, with an IC_50_ value of 0.96 ± 0.03 mg mL^−1^ (corresponding to 1.40 μg mL^−1^ of quercetin), while the control sample showed no relevant scavenging activity. As reported for the NO• scavenging activity, a comparison with the IC_50_ value of free quercetin (1.32 μg mL^−1^) also confirms that the antioxidant properties of the flavonoid were retained after the conjugation process.

Peroxynitrite radical was synthesized according to the literature [34], and fluorescein was used as ONOO^−^ detecting agent because it is bleached by hydroxyl, peroxyl radicals and by peroxynitrite and hypochlorite. In all cases, the bleaching can be prevented by antioxidants, so the degree of protection may be a measure of the antioxidant activity of a sample. pHEMA-Q was found to be a good protecting agent because of the ONOO^−^ scavenging, and the IC_50_ was found to be 2.93 ± 0.5 mg mL^−1^ corresponding to 4.28 μg mL^−1^ of quercetin. The inhibitory profile is reported as a function of the polymer concentration (Figure 5B), and the scavenging effect can be ascribed to the presence of quercetin in the polymeric chain; blank pHEMA, indeed, was not effective. The IC_50_ of free quercetin in the same reaction conditions was 1.12 μg mL^−1^ and the difference between the efficiency of free and bound Q could be ascribed to a variation of the scavenging mechanism moving from the two forms of Q. For the preparation of the pHEMA-Q conjugate, the employed synthetic procedure does not consist of a common derivatization process, but of a linkage of quercetin (through the aromatic ring or OH groups) to the HEMA growing chains by a radical mechanism. As reported in literature [46], among the flavonoids, a modification of their electronic distribution can strongly modify the peroxynitrite scavenging activitiy.

A further characterization of the antioxidant properties was performed in terms of lipid peroxidation inhibition (Figure 4C). Linoleic acid was used as a substrate and β-carotene as detecting agent. In the β-carotene-linoleate model system, one hydrogen atom of linoleic acid is withdrawn, leaving a free radical ready to attack β-carotene molecules. In this reaction, β-carotene molecules lose the double bond and their characteristic orange color fades, and this oxidative degradation of β-carotenes by radicals on linoleic acid is measured by the decrease in absorbance at 470 nm [33]. The inhibition of the lipid peroxidation by different concentrations of the pHEMA-Q is reported in Figure 5C. The high antioxidant properties of the conjugate are clearly understandable, with an IC_50_ value of 2.63 ± 0.2 mg mL^−1^. Also in this case, the blank pHEMA was ineffective. The recorded IC_50_ value corresponds to a 3.84 μg mL^−1^ Q concentration, quite similar to that of free Q (3.10 μg mL ^−1^), showing that the conjugation did not negatively interfere with this antioxidant ability.

Another key activity of an antioxidant material is its ability to inhibit the hydroxyl radical formation. Hydroxyl radicals, indeed, exhibit very high reactivity and tend to react with a wide range of molecules found in living cells. They can interact with the purine and pyrimidine bases of DNA. They can also abstract hydrogen atoms from biological molecules (e.g., thiol compounds), leading to the formation of sulfur radicals able to combine with oxygen to generate oxysulfur radicals, a number of which damage biological molecules [35]. Due to the high reactivity, the radicals have a very short biological half-life. Thus, an effective scavenger must be present at a very high concentration or possess very high reactivity toward these radicals. Although hydroxyl radical formation can occur in several ways, by far the most important mechanism *in vivo* is the Fenton reaction where a transition metal is involved as a pro-oxidant in the catalyzed decomposition of superoxide and hydrogen peroxide. These radicals are intermediary products of cellular respiration, phagocytic outburst and purine metabolism. Hydroxyl radical can be generated *in situ* by decomposition of hydrogen peroxide by high redox potential EDTA–Fe^2+^ complex, and in the presence of deoxyribose substrate, it forms thiobarbituric acid-reactive substances (TBARS) which can be measured. Antioxidant activity is detected by decreased TBARS formation, which can come about by donation of hydrogen or electron from the antioxidant to the radical or by direct reaction with it. Consequently, the ability of the synthesized polymers to scavenge hydroxyl radical was evaluated by the Fenton-mediated deoxyribose assay. Also this test confirmed the good antioxidant properties of functional materials compared to blank pHEMA (Figure 5D), with an IC_50_ value of 2.20 ± 0.4 mg mL^−1^. As reported for the lipid peroxidation assay, this value (3.21 μg mL^−1^ quercetin equivalent concentration) is similar to the IC_50_ value of free Q (2.98 μg mL^−1^).

## 4. Conclusions

In this work, a new functional biomaterial for application in ophthalmology was synthesized by free radical grafting reaction of 2-hydroxyethyl methacrylate (HEMA) and quercetin. The reaction mechanism involves the direct insertion of the antioxidant quercetin in the growing polymeric chain of HEMA. Instrumental analyses, such as FT-IR and UV-Vis analyses, were used as a confirmation of the presence of the antioxidant in the final material, while the Folin-Ciocalteu, as a crude estimation of the functionalization degree, was expressed as mg of Q bound per g of dry polymer. After these preliminary characterizations, the biocompatibility of the conjugate was explored by the HET-CAM test, resulting in an irritancy index of zero (high compatibility and no irritant effects). The conjugate could be applied for the preparation of innovative contact lenses: the presence of quercetin, indeed, confers high antioxidant and anti-inflammatory activities to pHEMA, with an improvement of its properties, thus the potential applicability of this polymeric material in ophthalmology is significantly improved.
